# Revisional Endoscopic Foraminal Decompression via Modified Interlaminar Approach at L5-S1 after Failed Posterior Instrumented Lumbar Fusion in Elderly Patients

**DOI:** 10.3390/bioengineering10091097

**Published:** 2023-09-19

**Authors:** Zheng Cao, Zhenzhou Li, Hongliang Zhao, Jinchang Wang, Shuxun Hou

**Affiliations:** Senior Department of Orthopedics, The Fourth Medical Center of PLA General Hospital, Beijing 100048, China; caozheng_81@163.com (Z.C.); njhq121@163.com (H.Z.); dcjinchangwang@126.com (J.W.); hsxortho@yeah.net (S.H.)

**Keywords:** endoscopic spine surgery, revision, arthrodesis surgery, foraminal stenosis, interlaminar, elderly patients

## Abstract

Elderly people usually have poorer surgical tolerance and a higher incidence of complications when undergoing revision surgery after posterior instrumented lumbar fusion (PILF). Full-endoscopic transforaminal surgery is a safe and effective option, but sometimes, it is difficult to revise L5-S1 foraminal stenosis (FS) after PILF. Therefore, we developed full-endoscopic lumbar decompression (FELD) at the arthrodesis level via a modified interlaminar approach under local anesthesia. This study aimed to describe the technical note and clinical efficacy of the technique. Eleven patients with unilateral lower limb radiculopathy after PILF underwent selective nerve root block and then underwent FELD. Magnetic resonance imaging (MRI) and computer tomography (CT) were performed on the second postoperative day. Their clinical outcomes were evaluated with a Visual analog scale (VAS) of low back pain and sciatica pain, Oswestry disability index (ODI), and the MacNab score. Complete decompression was achieved in every case with FELD without serious complications. Postoperative VAS of sciatica pain and ODI at each time point and VAS of low back pain and ODI after three months postoperatively were significantly improved compared with those preoperative (*p* < 0.05). According to the MacNab criteria, seven patients (63.6%) had excellent results at the two-year follow-up, and four patients (36.4%) had good results. No patients required further revision surgery. FELD, via a modified interlaminar approach, is effective for treating unilateral L5-S1 FS after PILF in elderly people.

## 1. Introduction

Spinal arthrodesis surgery is usually used for patients with traumatic or degenerative spinal disease in whom conservative treatments fail. Since the development of screw fixation by Michele and Krueger in 1949 and metallic rod stabilization by Harrington in 1953, internal fixation techniques for promoting bone fusion by instrumentation have advanced rapidly [1]. The number of patients with failed back surgery syndrome (FBSS) increases as more and more lumbar surgeries are being performed, and many of them are unsuccessful [2]. The most common diagnoses of FBSS are foraminal stenosis (FS) (25% to 29%) [3]. The revision rates after posterior instrumented lumbar fusion (PILF) are increasing [4].

An FS with failed conservative treatment associated with FBSS often requires lumbar revision surgery, such as extensive foraminotomy and decompression. The success rate of revision surgery in patients with FBSS is low and declines with each subsequent procedure [5]. However, epidural fibrosis, scar tissues, and altered anatomy significantly increase surgical complications such as dural tears and neural injury. Elderly people usually have poorer surgical tolerance and a higher incidence of complications when undergoing revision surgery after PILF. The advent of minimally invasive spinal procedures, such as full-endoscopic spinal surgery, which does not require broad muscle stripping, posterior structure resection, or extensive exposure of neural elements of the lumbar spine, is related to a lower incidence of short- and long-term postoperative complications [6], many surgeons performed revision surgery following lumbar fusion surgery with transforaminal approach with excellent outcomes [6,7,8,9,10]. For elderly patients at high risk for general anesthesia, the full-endoscopic procedure via transforaminal approach under local anesthesia may be a good option. However, the patient’s anatomy, such as a high or narrow iliac crest or the extraordinarily large and overhanging L5 transverse processes, may limit the ability to enter the L5-S1 foramen [3]. The fusion instrument and posterolateral bone grafting may increase the difficulty of transforaminal decompression. Hence, transforaminal decompression can be challenging for revising L5-S1 FS after PILF.

The classic interlaminar approach is usually performed under general anesthesia [11], and it’s difficult to decompress the foramen and may result in segmental instability [12]. Interlaminar contralateral endoscopic lumbar foraminotomy has been reported with satisfactory results [13,14,15,16,17,18]. This approach might have more benefits at L5-S1 after PILF because of the anatomical features that may be limiting factors during the transforaminal endoscopic approach, such as high or narrow iliac crest, large and overhanging L5 transverse processes, and fusion instrument and posterolateral bone grafting. The operation was performed under local anesthesia, which is safer for elderly patients. Rapid osteotomy by removing the superior articular process (SAP) and partial lamina using a trephine system we designed via a modified interlaminar approach may reduce the operation time with sufficient decompression. This technology may provide a new option for the treatment of L5-S1 FS after failed PILF in the elderly.

In this study, full-endoscopic lumbar decompression (FELD) under local anesthesia via a modified interlaminar approach was performed to treat L5-S1 FS after PILF. We aimed to detail the technical note of the FELD procedure and assess the clinical efficacy and safety of this technology.

## 2. Materials and Methods

### 2.1. Patients, Inclusion & Exclusion Criteria

From January 2015 to December 2018, 11 consecutive patients over 60 years old with FS after failed PILF in our department were enrolled in the study using the following inclusion criteria:

(1) Clinical signs of unilateral lumbar monoradiculopathy after PILF;
(2) Concordant imaging evidence of monosegmental FS at the same level within the fusion segment demonstrated on lumbar magnetic resonance imaging (MRI) and/or computer tomography (CT) scans;
(3) Unsuccessful conservative treatment for at least 12 weeks;
(4) Patients who agreed to sign informed consent to participate in this evaluation and are willing to return for follow-ups.

The following exclusion criteria were employed to prevent patients from being enrolled into our study:

(1) Bilateral symptoms or involving more than one dermatome;
(2) Severe central stenosis on preoperative MRI or CT and/or Cauda equina syndrome;
(3) Other diseases that may cause similar neurological symptoms, such as peripheral neuropathy;
(4) Patients with systematic infection or bleeding diathesis difficult to improve;
(5) Patients with unrealistic expectations and/or uncooperative patients.

Approval to conduct this study was granted by the ethics committees of the Fourth Medical Center of PLA General Hospital. Institutional Review Board approved informed consent, and protocols were provided to all the patients, which described details of the surgery, including multiple treatment options, predictive outcome, potential risks, and side effects.

### 2.2. Preoperative Work-Up

**Figure 1:**
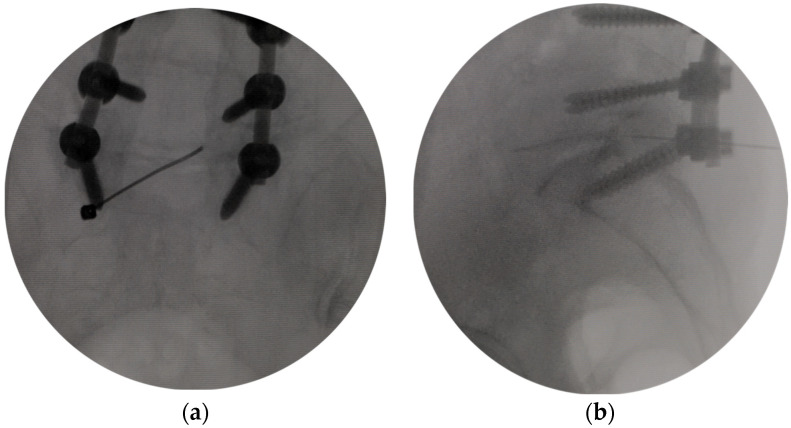
Selective nerve root block under fluoroscopic guidance. (**a**) The tip of the needle was located at the line of the medial border of pedicles in the fluoroscopic anteroposterior view. (**b**) The tip of the needle was located at the posterior inferior margin of the superior vertebral body in the fluoroscopic lateral view.

All patients underwent diagnostic selective nerve block before the operation to define the nerve root responsible for the patient’s persistent pain. Because of FS and the obstruction of internal fixation and bone graft, the L5 nerve block was difficult to perform through the transforaminal approach. Therefore, all selective nerve blocks were performed through an interlaminar approach. Patients were positioned prone on a radiolucent table. A 20-G Tuohy needle was introduced into the midline at the level of the pathology and was advanced toward the superomedial margin of the interlaminar foramen until it touched the bone. The needle was pushed forward along the bone until the tip was located at the line of the medial border of the pedicles in the fluoroscopic anteroposterior (AP) view (Figure 1a) and the inferior posterior margin of the superior vertebral body in the fluoroscopic lateral view (Figure 1b). After confirming the final position of the needle tip, 2 mL of Ioversol was injected, and the diffusion range of the contrast agent was evaluated. Then 1 mL of 0.5% lidocaine and 1 mL (40 mg) triamcinolone acetonide were injected into the epidural space. The injection was deemed diagnostic if the typical pain could be reproduced with the advancement of the needle and initial injection of contrast agent consistent with the corresponding dermatome of the symptomatic nerve root and pain relief within 2 h after the injection while asking the patient to perform the provocative maneuver or activity that typically would elucidate their familiar pain.

### 2.3. Surgical Procedures

**Figure 2:**
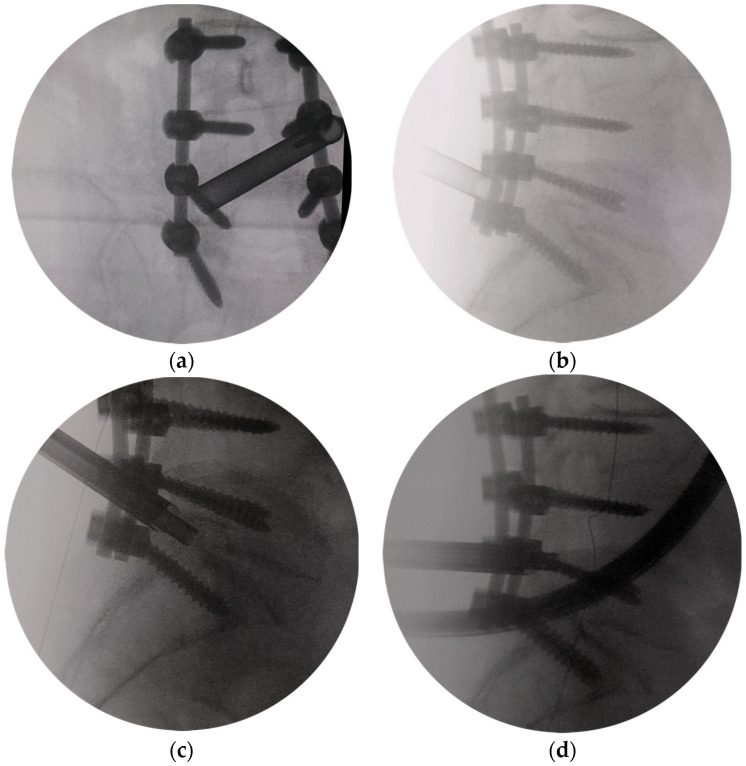

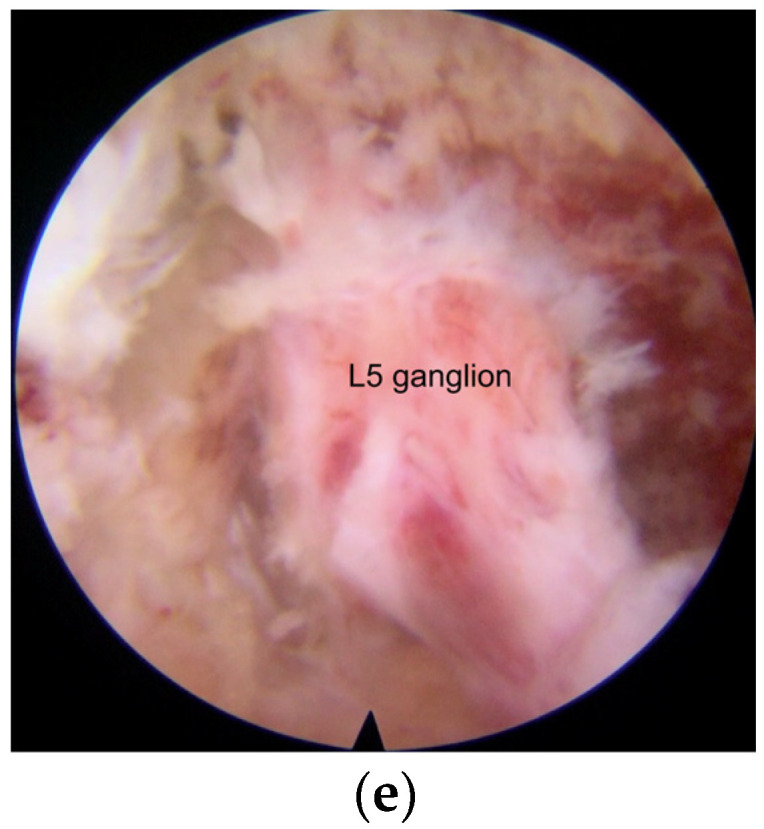
The demonstration of a full-endoscopic decompression procedure via a modified interlaminar approach. (**a**) The tip of the protective sleeve was located at the line of the medial border of pedicles in the fluoroscopic anteroposterior view. (**b**) The tip of the protective sleeve was located at the dorsal of the intervertebrale foramen in the fluoroscopic lateral view. (**c**) Trephine was used to resect part of the S1 superior articular process. (**d**) Trephine was used to resect the dorsal part of the L5 lateral recess. (**e**) Enlarged intervertebral foramen under Endoscopic view. L5 nerve root and ganglion were adequately decompressed. The shoulder, the axilla, and the dorsal aspect of the exiting nerve root were viewed and adequately decompressed.

All the patients were operated on under conscious sedation and analgesia of local anesthesia with 0.5% lidocaine. In every procedure, a single-shot second-generation cephalosporin was applied intravenously preoperatively. All patients were placed in the prone position on a radiolucent table with C-arm imaging. The surgeon stayed on the side opposite to that of the FS. After the interlaminar window of the pathology was identified, a 1 cm long longitudinal skin incision was made. A dilator was inserted bluntly into the superolateral margin of the interlaminar window. The trajectory could be defined on preoperative MRI/CT and C-arm AP view. A 7 mm working cannula was passed over the obturator and advanced. After confirming the final position of the cannula tip in AP (Figure 2a) and lateral view (Figure 2b), a trephine [19], designed by us, was advanced with careful rotation to remove the central part and the tip of S1 SAP (Figure 2c). Then, the medial and ventral aspects of the L5 inferior articular process (IAP) were removed using the trephine. Finally, the dorsal part of the L5 lateral recess was removed (Figure 2d). The cannula tip position was always checked in lateral view after the trephine had been moved. A 25° endoscope (Spinendos GmbH, München, Germany) with a working channel of 4.3 mm and a working length of 130 mm was inserted.

Further decompression was performed under the endoscope view with endoscopic Kerrison punch, drill (Spinendos GmbH, München, Germany), or ultrasonic osteotome (SMTP, Beijing, China). The lateral foramen was decompressed by removing the medial part of the facet and undercutting the lateral aspect of the facet until the outer lateral part of the foramen was seen. Then, the soft scar tissue was accessible to be detached from the bone. The space between the L5 and S1 pedicles on the symptomatic side was visualized. The origin of the exiting nerve root was revealed by sometimes removing the dorsal part of the L5 lateral recess and the medial and inferior parts of the L5 pedicle. The lateral aspect of the dura and the dorsal aspect of the L5 nerve root and dorsal root ganglia (DRG) were exposed. The space underneath the root was probed with a small hook dissector. Finally, the shoulder, the axilla, and the dorsal aspect of the L5 nerve root were adequately decompressed (Figure 2e). Pressure could be changed by the intermittent block of the irrigation saline outflow with the thumb, allowing the exiting nerve root to move freely, confirming complete decompression. After adequate hemostasis with a bipolar radiofrequency electrode (Elliquence LLC, New York, NY, USA), the endoscope was withdrawn, and the incision was sutured subcutaneously with an absorbable suture.

### 2.4. Postoperative Care

**Figure 3:**
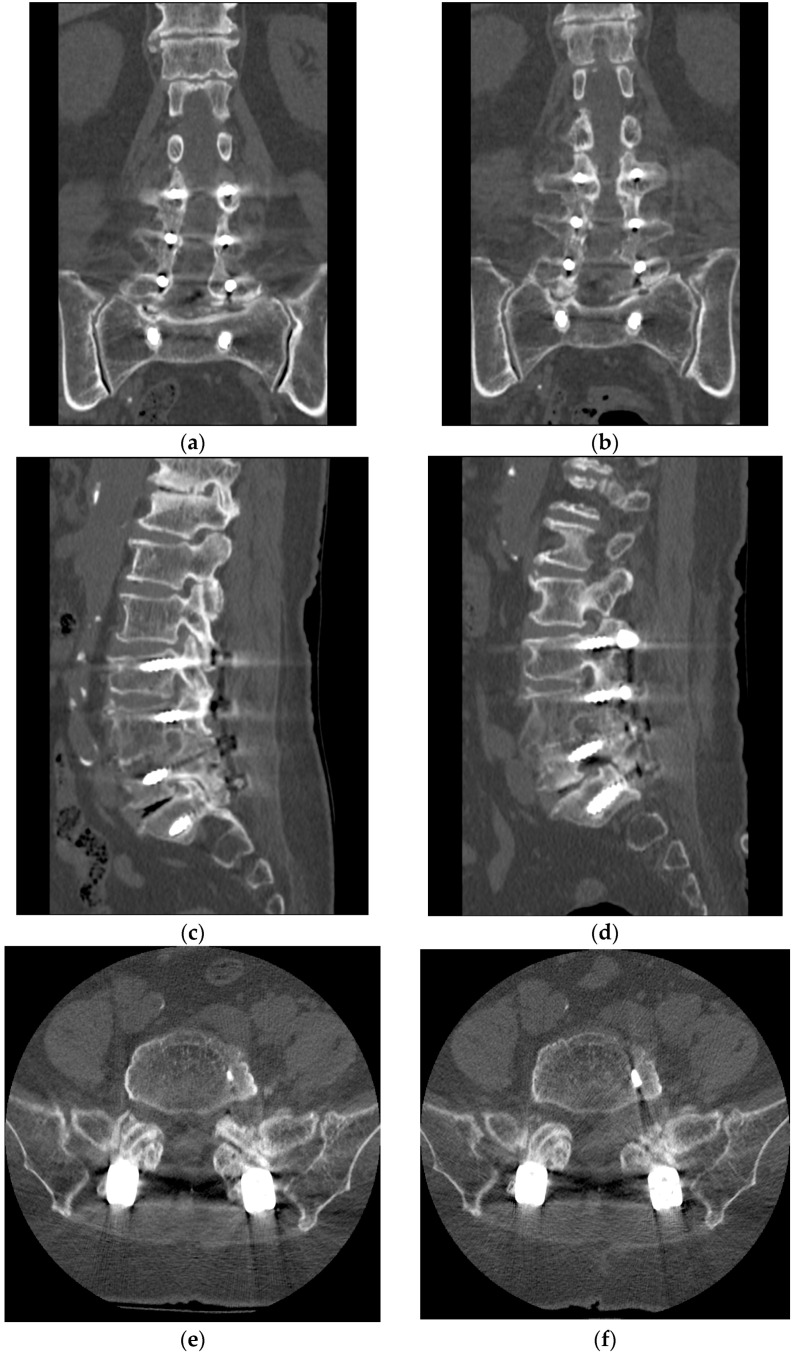

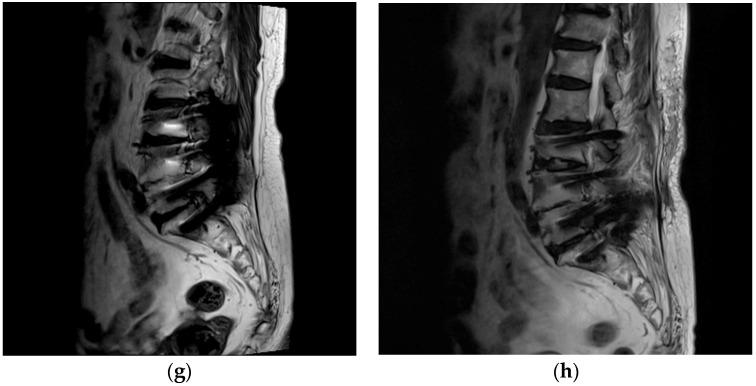
Preoperative and postoperative computed tomography and magnetic resonance images. (**a**) Preoperative coronal CT image showed that the effect of L5 pedicular kinking may be one of the reasons for the high tension of the L5 nerve root. (**b**) Postoperative coronal CT image showed partial pedicle excision to ensure adequate shoulder decompression of the L5 root. (**c**) Preoperative sagittal CT showed L5-S1 intervertebral foramen stenosis attributed to compression between the S1 superior articular process and the posterolateral osteophytes from the L5 vertebral endplate. (**d**) Postoperative sagittal CT showed enlarged L5-S1 intervertebral foramen after excision of the S1 superior articular process tip. (**e**) Preoperative axial CT showed the stenosis of the entrance zone of the left L5-S1 intervertebral foramen caused by the hyperplasia of the S1 superior articular process. (**f**) Postoperative axial CT showed enlarged L5-S1 intervertebral foramen by resecting the S1 superior articular process tip. (**g**) Preoperative MRI showed that the L5 nerve root was compressed at the L5-S1 intervertebral foramen. (**h**) Postoperative MRI showed the enlarged intervertebral foramen and the edema but uncompressed L5 nerve root.

All the patients were mobilized the morning after surgery and discharged after postoperative MRI and CT performed one day after surgery (Figure 3). A lumbar brace during mobilization was prescribed for about four weeks. Some exercises, including isometric and coordinative exercises, were guided by rehabilitation doctors before surgery and were performed without supervision after surgery. No physical therapy was prescribed. Oral administration of meloxicam as a 7.5 mg tablet for one week was prescribed. Symptom relief was evaluated by follow-up interviews at preoperative and day one, three months, six months, 12 months, and 24 months after surgery. Low back and sciatica pain was measured using a visual analog scale (VAS) score (1–100). Functional outcomes were assessed by using the Oswestry Disability Index (ODI) (preoperative, three months, six months, 12 months, and 24 months after surgery) and modified MacNab criteria (2-year follow-up). The preoperative and postoperative heights and widths of the pathological foramina and the posterior disc heights at L5–S1 were obtained from sagittal CT views [20]. Statistical analyses were performed with Statistical Package for Social Sciences 26 software (IBM Corp., Chicago, IL, USA). Preoperative and postoperative VAS scores of low back sciatica pain and ODI values were analyzed with ANOVA. Preoperative and postoperative heights and widths of the pathological foramens were analyzed using Student’s *t* test. *p* < 0.05 was considered a significant difference.

## 3. Results

Eleven patients treated with FELD at the arthrodesis level via a modified interlaminar approach were included in this study, including four females and seven males. The average age was 69.3 years (60–78 years). The number of instrumented fused levels was two segments in two patients (18.2%), three segments in four patients (36.4%), four segments in three patients (27.3%) and five segments in two patients (18.2%). Total laminectomy was performed in all the fused levels. Single cage intervertebral fusion was performed at the L5-S1 segment in 4 cases, and posterolateral fusion without intervertebral fusion was performed at the L5-S1 segment in 7 cases. The main symptoms of the patients were relieved after the previous PILF surgery. Clinical signs of unilateral lumbar mono-radiculopathy after PILF appeared at an average of 2.9 years (1.5–6.5 years) after surgery. Preoperative CT images showed that foraminotomy of the index level had not been performed during the previous PILF surgery. Preoperative CT showed S1 screw loosening in 9 cases and cage subsidence in all 4 cases with cage fusion. The average posterior disc height at L5–S1 was 2.7 ± 1.8 mm.

**Figure 4:**
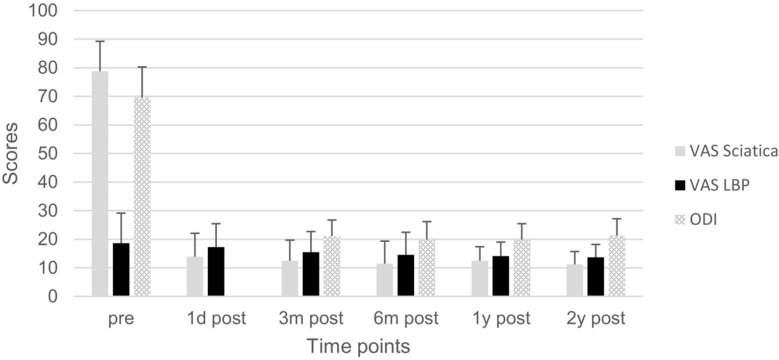
Clinical outcomes. Visual analog scale (VAS) scores for sciatica pain and low back pain preoperatively and at one day, three months, six months, one year, and two years postoperatively. Oswestry disability index (ODI) preoperatively and at three months, six months, one year, and two years postoperatively.

All operations were completed successfully. The average operation time was 45 min (30–80 min). There were no complications such as nerve injury, dural tear, nerve root function injury exacerbation, and infection among the cases. Postoperative CT showed that all the index intervertebral foramina were significantly enlarged (*p* < 0.05), with the height and width of the pathological foramen enlarged from 4.3 ± 0.7 mm and 3.9 ± 0.7 mm to 12.4 ± 1.4 mm and 20.3 ± 2.2 mm respectively. The perineural spaces surrounding the nerve root were visible, and no evidence of nerve root collapse or morphologic change was seen on postoperative MRI. Postoperative VAS of sciatica pain and ODI at each time point and VAS of low back pain and ODI after three months postoperatively were significantly improved compared with those preoperative (*p* < 0.05) (Figure 4). In addition, the MacNab score at the two-year follow-up indicated 7 cases (63.6%) with excellent and 4 cases (36.4%) with good MacNab outcomes. No Internal fixation fracture was found during the follow-up. No patients need further revision surgery.

## 4. Discussion

Cumulative reoperation rates after instrumented lumbar fusion were 12.5% at two years and 19.3% at four years, as Irmola T. et al. reported [4]. They showed an increase over time [21,22]. There are various strategies for revision surgery according to the underlying etiology. Complete decompression of the target nerve root in a narrowed lumbar foramen within an arthrodesis construct may result in symptomatic relief. Traditional methods may need to reopen the original incision, remove the existing internal fixation, and do a foraminotomy or total facetectomy. It may cause additional bleeding, infection, dural tear, and nerve injury. To improve outcomes, minimally invasive methods may be used to operate FBSS after PILF. Full-endoscopic spine surgerys show comparable clinical results to conventional open microsurgery with minor tissue trauma, shorter operation time, and faster recovery [23]. Since the technique of full-endoscopic lumbar foraminoplasty for various conditions of FBSS in selected cases was described by Knight et al. [24,25,26], the foraminal decompression technique with the transforaminal or extraforaminal approach has been developed for foraminal or extraforaminal stenosis. The full-endoscopic spine surgery via transforaminal approach effectively treats patients with continued back and sciatica pain after lumbar fusion due to FS, residual/recurrent nucleus pulposus, and pseudarthrosis [6,7,8,10,27]. However, endoscopic transforaminal decompression is complex in the case of L5-S1 FBSS at a previously instrumental fusion level because of the obstruction of the iliac crest, transverse processes of L5, posterolateral bone graft, hypertrophic facet, screw, and rod. The classic endoscopic interlaminar approach may have limitations in decompressing the nerve root at the lateral of the mid zone and exit zone as the lateral lumbar spinal canal stenosis classification by Lee et al. [28] because of the obstruction of the screw and rod. Interlaminar contralateral endoscopic lumbar foraminotomy for intraforaminal lumbar degenerative disease has been reported with successful results [13,14,15,16,17,18]. However, FELD at the arthrodesis level via an oblique interlaminar approach under local anesthesia in elderly patients with unilateral L5-S1 FS has not been reported.

**Figure 5:**
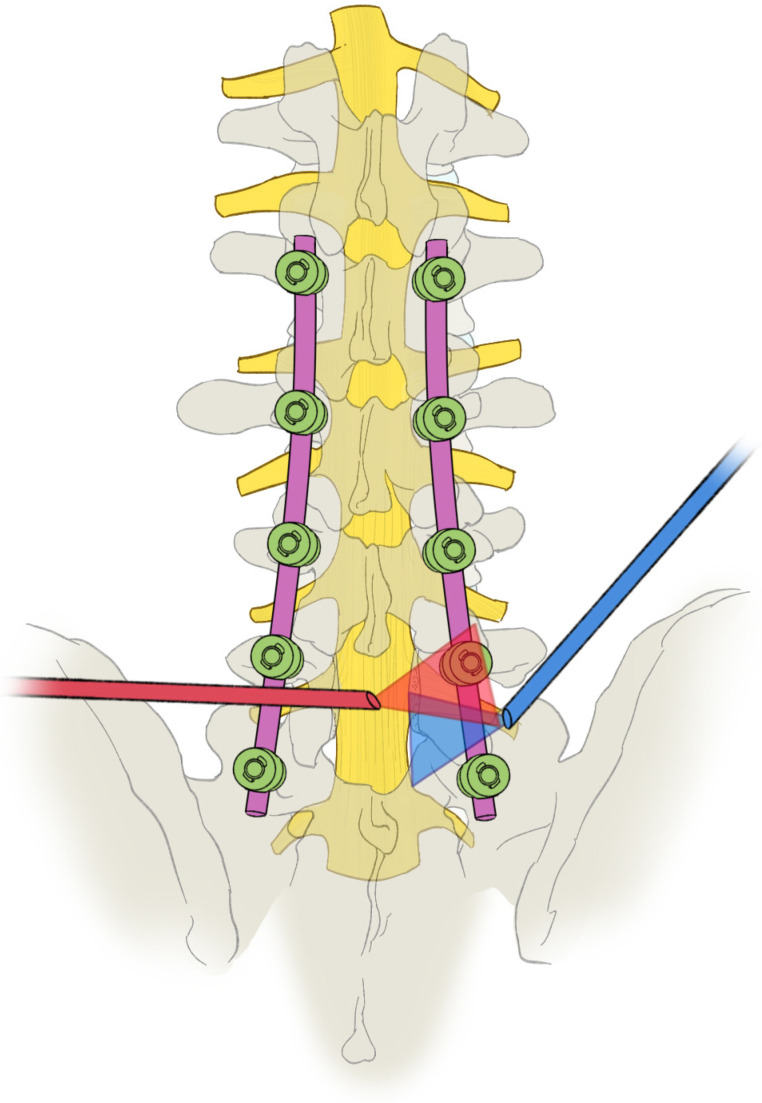
Schematic diagram of decompression range of transforaminal approach and modified interlaminar approach. The axilla area between the traversing and exiting nerves (Yellow) can be viewed and adequately decompressed with a transforaminal approach (Blue). The nerve root in both the entrance zone, mid zone and exit zone can be viewed and adequately decompressed with the modified interlaminar approach (Red).

The tip of a retained SAP left during the previous surgery was removed first in our operative technique. It frequently contributes to unrecognized residual stenosis in many cases of failed surgery [3]. The subluxation of the S1 SAP secondary to the loss of disc space height diminished the area and height of the L5-S1 foramen [29]. The insufficient surgical decompression in the index foraminal area during initial surgery may be one of the reasons for the occurrence of L5-S1 FS. The disc height is frequently decreased after interbody fusion [30]. Decreased disc height, especially the posterior disc height, leads to decreased foramen height, which can lead to significant exiting nerve root compression [31]. Decompression of the exiting nerve root requires partial or complete resection of the remaining S1 SAP to enlarge the intervertebral foramen. The SAP can also be partially resected, and the axilla area between the traversing and exiting nerves can be viewed and adequately decompressed with a transforaminal approach [3]. However, the shoulder of the exiting nerve between the lamina and pedicle is challenging to visualize or probe with the transforaminal approach to judge the adequacy of the decompression (Figure 5). Additional “double crush” adjacent and the intracanal stenotic lesion may exist, and it is sometimes difficult to determine the degree of this lesion involved in the pathology [31]. Pedicular kinking may cause severe root tethering, sometimes requiring partial pedicle excision to ensure adequate root decompression [31]. The L5 nerve root is more oblique, increasing its susceptibility to pedicular kinking and FS [32]. Volumetric reduction of the neuroforamen can also be created by the overgrown posterolateral fusion and thereby cause symptoms [6]. The SAP, the medial and inferior parts of the superior pedicle, foraminal osteophytosis, and herniated nucleus pulposus can be easily removed with the modified interlaminar approach, and the nerve root in both the entrance zone, mid zone, and exit zone can be viewed and adequately decompressed (Figure 5) [28]. Therefore, sufficient decompression of the nerve root is the key to achieving satisfactory surgical results.

Transient postoperative dysesthesia is the most common complication in the treatment of FS via transforaminal approach, with an incidence of 10.83% [33]. Postoperative dysesthesia and decreased motor function may occur because the L5 nerve root and DRG may be irritated during the L5-S1 transforaminal approach [23]. The L5 DRG, composed of sensory neurons sensitive to mechanical pressure, is commonly located in the foramen [34]. Any manipulation of the L5 DRG should be minimized or avoided because of the risk of worsening or development of pain and/or dysesthesia. Anthony Yeung [3] reported dysesthesia in 4 of 30 patients treated by endoscopic foraminal decompression for FBSS, which took 2–4 months for resolution. Two patients (2/20) in the microendoscopic discectomy group and two patients (2/28) in the percutaneous endoscopic lumbar discectomy group experienced dysesthesia after revision surgery for recurrent herniation in Yuan Yao’s study [35]. Jian-Jun Wu reported one case (1/16) of leg numbness caused by DRG injury in transforaminal endoscopic discectomy and foraminoplasty after lumbar fusion [27]. In comparison, DRG injury was not observed in our study. The potential reasons may be as follows:

(1) Because of the preexisting internal fixation, there is no concern for iatrogenic instability, allowing more extensive decompression and enlargement of the intervertebral foramen to avoid irritating the DRG.
(2) Under the endoscopic view, the structure of the foramen can be seen more clearly. Smaller operating instruments used in endoscopic operation can avoid irritating DRG.
(3) The modified interlaminar approach we used can show an almost parallel trajectory to the L5 nerve root in the foramen and provide excellent visualization of the nerve root all along its course without the need for significant retraction. This technique was called “no touch decompression” [36], as FS can be treated without more retraction of DRG compared with that via transforaminal approach, especially in complicated cases and L5-S1 cases with anatomical limitations [37].
(4) All operations were performed by a senior surgeon with extensive experience with the endoscopic operation. There is evidence that a lower incidence of complications has been observed when the contralateral interlaminar foraminotomy was performed by experienced surgeons [38].

Avoiding any manipulation of DRG as much as possible is the key to reducing postoperative neurological complications such as transient paralysis.

Dural tears and neural injury are frequent severe complications of spinal surgery. The risk of dural tears is higher when more power instruments are used to treat spinal stenosis via interlaminar approach [39]. A higher rate has been reported during the revision procedures because of the anatomical complexity, additional operation on instruments and bone grafts, nerve tissue adhesions, and scar tissue due to previous PILF [40]. Universally, adhesions and scar tissues are present between the neural elements, the ventral annulus, and surrounding tissues in revision surgery. It is difficult to develop an area between them, and there is a risk of dural tear and neural injury [41]. However, durotomies were not encountered in our study. The potential reasons may be as follows:

(1) Dissecting scar tissue from bone rather than nerve tissue is an effective method to reduce the risk of dural tear [42]. Bone tissue at the lateral and dorsal sides of the nerve structure was treated after the tip of the trephine, and anatomical landmarks were identified in X-ray view. The unscarred virgin tissue can be easily viewed and entered by endoscopic. Therefore, the operation can be safely performed without excessive retraction of the dura, nerve root, and DRG, which may be tethered at the foramen, and dissection of the scar tissues surrounding the nerve structure.
(2) The entry direction of the instrument is not toward the nerve root and dural sac. The operation in the spinal canal is far from the dural sac and nerve root, and the operation in the intervertebral foramen is parallel to the nerve root. It reduces the risk of dural tear and neural injury.
(3) Even if cerebrospinal fluid leakage occurs in endoscopic surgery, it is not easy to find it. There is usually no need for special treatment, and it does not affect postoperative recovery [39].
(4) The number of cases is small. The incidence of neural injury may increase with higher caseloads, but it may be low.

Starting an operation from decompression of bone structure and avoiding excessive manipulation of scar tissue may be a way to avoid such complications.

There were a few limitations in this study. First, due to the low incidence of unilateral L5-S1 FS after PILF, the sample size was small. Second, it was just a retrospective study. Third, the relatively short follow-up to its long-term efficacy needs time to verify. With the extension of follow-up, patients may suffer from some symptoms or complications that require revisional surgeries. Therefore, the efficacy and safety of FELD via a modified interlaminar approach in the treatment of L5-S1 FS after PILF have yet to be confirmed by the results of large-scale, long-term follow-up, prospective controlled studies.

## 5. Conclusions

Full-endoscopic foraminal decompression via a modified interlaminar approach under local anesthesia is effective for treating unilateral L5-S1 FS after PILF in elderly patients. The modified interlaminar approach can obtain complete decompression with good clinical outcomes.

## Data Availability

The datasets analyzed during the current study are available from the corresponding author upon reasonable request.
